# Supplementary material for: Increasing the Grain Yield and Grain Protein Content of Common Wheat (Triticum aestivum) by Introducing Missense Mutations in the Q Gene
Source: Int J Mol Sci. 2022 Sep 15;23(18):10772. doi: 10.3390/ijms231810772 (PMC9505668; doi:10.3390/ijms231810772)
Supplement: Supplementary file 1 [file ijms-23-10772-s001.zip › ijms-1912404-supplementary.pdf]

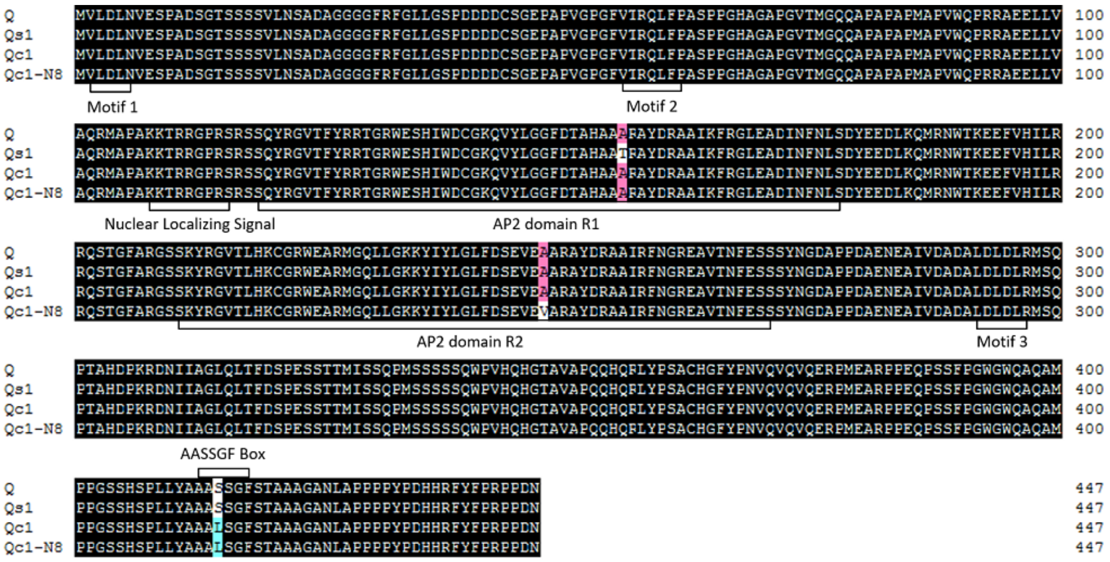

Figure S1. Alignment of the deduced amino acid sequences encoded by the *Q*, *Q*^{s1}, *Q*^{c1}, and *Q*^{c1}*-N8* alleles. Seven previously described conserved domains (motif 1, motif 2, nuclear localization signal, AP_2 domain R1, AP_2 domain R2, motif 3, and AASSGF box) are presented.

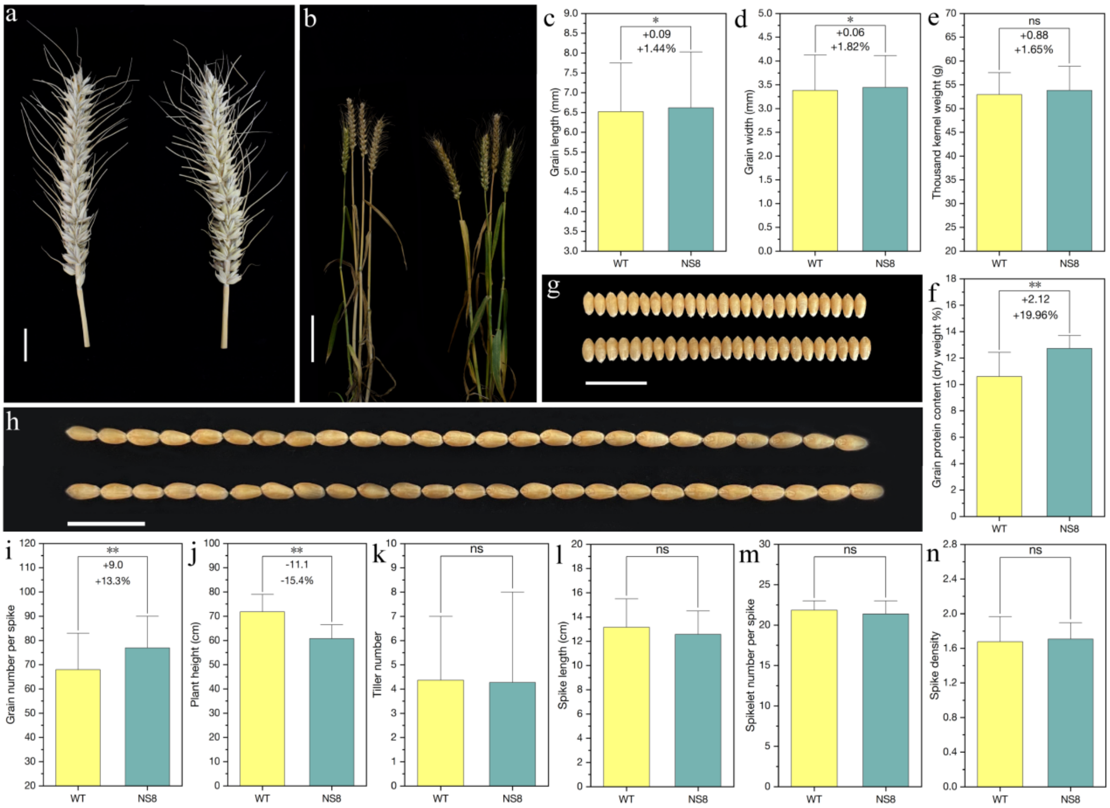

Figure S2. Effect of Q^{c1}-*N8* on agronomic traits during the 2021-2022 growing season. (a) Spikes of the wild-type (WT) (left) and *NS8* (right) plants. Scale bar, 1 cm. (b) WT (left) and *NS8* (right) plants. Scale bar, 10 cm. Comparisons of the grain length (c), grain width (d), thousand kernel weight (e), grain protein contents (f), grain number per spike (i), plant height (j), tiller number (k), spike length (l), spikelet number per spike (m), and spike density (n) of the WT and *NS8* samples. **, $P < 0.01$; *, $P < 0.05$; ns, not significant. Data are presented as the mean ± standard deviation. '+' and '–' indicate more and less than the WT control, respectively. '+ 0.09' in panel (c) indicates the *NS8* grain was 0.09 mm longer than the WT grain (on average). '+ 1.44%' in panel (c) indicates that Q^{c1}-*N8* increased the grain length by 1.44% (on average). (g) Kernel width of the WT (upper) and *NS8* (lower) samples. Scale bar, 2 cm. (h) Kernel length of the WT (upper) and *NS8* (lower) samples. Scale bar, 2 cm.
